# Ultrasonographic assessment of cervical and craniofacial muscle thickness in individuals with and without cervicogenic headache

**DOI:** 10.7717/peerj.21285

**Published:** 2026-05-25

**Authors:** Hatice Ağır, Mehmet Göktepeli

**Affiliations:** 1 Department of Physical Medicine and Rehabilitation, Şanlıurfa Training and Research Hospital, Şanlıurfa, Şanlıurfa, Turkey; 2 Department of Physical Medicine and Rehabilitation, Private Koru Hospital, Ankara, Ankara, Turkey; 3 Department of Radiology, Şanlıurfa Education and Research Hospital, Şanlıurfa, Turkey

**Keywords:** Cervicogenic headache, Neck pain, Ultrasonography, Rehabilitation, Muscle morphology

## Abstract

**Background:**

Cervicogenic headache (CH) is a secondary headache disorder originating from dysfunction of the cervical spine and surrounding musculoskeletal structures. Alterations in cervical and craniofacial muscle morphology may be related to its pathophysiology. This study aimed to compare the thickness of selected head and neck muscles between individuals with and without CH using ultrasonography and to examine potential associations with clinical symptoms.

**Methods:**

A prospective cross-sectional study was conducted with 37 patients diagnosed with CH and 37 age-and sex-matched healthy controls. The thickness of the sternocleidomastoid, upper trapezius, masseter, and temporalis muscles was bilaterally measured using portable ultrasonography. Headache intensity and functional disability were assessed with standardized clinical instruments. Group comparisons and correlation analyses were performed using appropriate statistical methods.

**Results:**

Participants with CH demonstrated significantly reduced thickness in the bilateral sternocleidomastoid and right upper trapezius compared with controls. No significant differences were observed in the masseter or temporalis muscles. A moderate negative correlation was found between right trapezius thickness and disability scores, whereas headache intensity was not significantly related to muscle thickness.

**Conclusions:**

Sternocleidomastoid and upper trapezius thinning may be associated with cervicogenic headache. Ultrasonography provides a non-invasive tool for evaluating muscle morphology in this population; however, further studies are needed to clarify its clinical implications and potential role in guiding rehabilitation approaches.

## Introduction

Cervicogenic headache (CGH) is a secondary headache disorder arising from dysfunction of the cervical spine and its associated musculoskeletal structures (International Headache Society (IHS), 2018). Clinically, CGH is typically characterized by unilateral pain that originates in the occipital region and radiates toward the frontal or temporal areas, often aggravated by neck movements (Fernández-de-las-Peñas & Cuadrado, 2014). Its prevalence in the general population ranges from 0.4% to 2.5%, and it accounts for approximately 14–18% of all chronic headaches (Biondi, 2005). The underlying neuroanatomical mechanism of CGH is thought to involve the convergence of afferent fibers from the upper cervical spinal nerves (C1–C3) and the trigeminal nerve within the trigeminocervical complex. This neuroanatomical convergence enables the referral of nociceptive input between cervical and trigeminal territories, contributing to head and facial pain (Edvinsson et al., 2020). Persistent nociceptive input from cervical structures may also lead to alterations in neuromuscular control, including modified muscle activation patterns and impaired sensorimotor function in the cervical region (Falla & Farina, 2005; Lin et al., 2022). These neuromotor adaptations may, over time, be accompanied by measurable changes in muscle morphology.

Research has increasingly focused on structural and functional alterations in cervical muscles in CGH. Ultrasonographic studies have demonstrated reduced thickness and cross-sectional area of deep cervical flexor muscles, particularly the longus colli, as well as altered activation patterns and decreased endurance capacity in individuals with cervicogenic headache (Abaspour et al., 2015; Abaspour, Akbari & Rezasoltani, 2020; Jull et al., 2007). In addition to reduced muscle thickness, changes such as impaired neuromuscular control, decreased proprioceptive acuity, and altered motor coordination strategies have been reported in patients with recurrent headache disorders. Furthermore, age-related structural alterations, including reduced muscle mass and possible fatty infiltration in cervical muscles, have been observed in elderly women with CGH (Uthaikhup et al., 2017).

However, most prior studies in CGH have primarily emphasized the role of deep cervical flexors (*e.g*., longus colli, longus capitis) and extensors (*e.g*., semispinalis, suboccipital muscles), as these muscles are strongly associated with sensorimotor control and cervical spine stability. In contrast, superficial cervical muscles such as the sternocleidomastoid (SCM) and upper trapezius (UT) have received relatively less attention, despite their clear biomechanical and postural relevance in maintaining head and neck alignment (Choi, 2021). The SCM and UT muscles play important roles in dynamic neck stabilization, scapulocervical rhythm, and postural control. Previous biomechanical and clinical studies have suggested that altered activation patterns or mechanical properties of these muscles may contribute to changes in load distribution and motor control strategies in individuals with neck pain and CGH (Sedlackova et al., 2022; Mohammadi et al., 2021).

The inclusion of craniofacial muscles such as the masseter and temporalis was intended to provide a broader assessment of head and neck muscle morphology in individuals with cervicogenic headache. Although nociceptive inputs from upper cervical structures converge with trigeminal afferents within the trigeminocervical complex (Edvinsson et al., 2020), CGH is primarily considered a cervical musculoskeletal disorder. Moreover, the masticatory muscles are motor-innervated by the mandibular branch of the trigeminal nerve rather than by cervical nerves. Therefore, in the absence of concomitant temporomandibular dysfunction, substantial morphological alterations in masseter and temporalis muscles would not necessarily be expected. Their evaluation in the present study was exploratory and aimed to determine whether any concurrent craniofacial muscle differences accompanied cervical muscle changes.

Ultrasonography (USG) offers a non-invasive, accessible, and reliable method for evaluating muscle morphology, including thickness and cross-sectional area. Compared with magnetic resonance imaging (MRI) or computed tomography (CT), which are limited by cost and accessibility, and surface electromyography (EMG), which may be influenced by adjacent muscle activity, US provides an objective morphological assessment (Sedlackova et al., 2022; Peterson et al., 2019).

Therefore, the aim of this study was to compare the thickness of the SCM, UT, masseter, and temporalis muscles in individuals with and without CGH using ultrasonography and to examine potential associations between muscle thickness and clinical symptoms. We hypothesized that individuals with CGH would show a reduction in the thickness of selected cervical muscles compared to a control group. Additionally, craniofacial muscles were evaluated to investigate the potential distribution of morphological differences beyond the cervical region.

## Materials and Methods

### Study participants

This prospective, cross-sectional study included a total of 74 participants, consisting of 37 patients diagnosed with CGH and 37 age-and sex-matched healthy controls. Patients were consecutively recruited from individuals who presented to the Physical Medicine and Rehabilitation outpatient clinic at Şanlıurfa Training and Research Hospital between April 2023 and January 2024 with complaints of chronic unilateral headache accompanied by cervical dysfunction.

The diagnosis of CGH was based on the International Classification of Headache Disorders, 3rd edition (International Headache Society (IHS), 2018) criteria, which require:

(1) Unilateral headache associated with cervical musculoskeletal dysfunction,
(2) Headache provoked or exacerbated by neck movement or sustained posture,
(3) Headache relief following diagnostic block or manual examination.

Because invasive diagnostic nerve blocks are not routinely feasible in daily clinical practice, diagnosis in this study relied on a standardized clinical assessment protocol performed by a board-certified physical medicine and rehabilitation specialist with >7 years of clinical experience in musculoskeletal pain and headache management. Diagnostic confirmation was made by reproducing headache during manual palpation of the upper cervical spine and paraspinal musculature, in accordance with prior validation studies supporting palpation as a reliable diagnostic adjunct in CGH (Jull et al., 2007).

The control group consisted of healthy volunteers recruited from hospital staff and patient relatives, with no history of headache, neck pain, cervical spine disorders, temporomandibular joint dysfunction, or neurological disease.

Exclusion criteria for both groups were: age <18 or >55 years, diagnosis of other primary headaches (*e.g*., migraine or tension-type headache), recent cervical trauma or surgery, systemic inflammatory or rheumatological disease, pregnancy, or migraine-related features such as photophobia, phonophobia, or aura.

All participants provided written informed consent prior to enrollment. The study protocol was approved by the Harran University Faculty of Medicine Ethics Committee (Approval No: HRÜ/23.05.05; date: 27.03.2023).

#### Ultrasonographic assessment

Muscle morphology was assessed using a GE Vscan Air^™^ portable ultrasound system (General Electric Healthcare, USA) equipped with a high-resolution linear probe (7–15 MHz). All measurements were performed by an experienced radiologist (M.G.) with >10 years of musculoskeletal imaging experience, who was blinded to group assignments to minimize observer bias.

Each muscle was examined bilaterally, and three repeated measurements were taken on each side. The mean of these three measurements was used for statistical analysis. To ensure consistency, participants were positioned to minimize involuntary muscle activation and were instructed to remain relaxed during imaging. Intra-rater reliability was tested in a random subsample of 10 participants, showing high reproducibility (intraclass correlation coefficient >0.85).

We included both cervical (SCM, UT) and craniofacial (masseter, temporalis) muscles in order to explore their potential contribution to CGH symptoms, based on their anatomical relevance and neurophysiological overlap in pain referral patterns rather than to represent the entire cervical musculature spectrum.

**Measurement protocols:**

- **SCM:** Participants were positioned supine with the head in a neutral position. The probe was placed transversely at the mid-segment of the SCM, along the line between the mastoid process and the sternoclavicular junction. Muscle thickness was defined as the maximum distance between the superficial and deep fasciae at the thickest cross-section.
- **UT:** Participants were seated with shoulders relaxed. The probe was placed at the midpoint between the acromion and the C7 spinous process, perpendicular to the muscle fibers, and thickness was measured as the maximum distance between the superficial and deep fascial layers.
- **Masseter:** Participants were positioned supine with the mandible in light occlusion. The probe was placed vertically over the belly of the masseter, lateral to the mandibular *corpus*. Thickness was defined as the maximum distance between the superficial and deep fasciae of the muscle.
- **Temporalis:** Participants were examined in a seated position. The probe was placed vertically over the temporal fossa, just above the zygomatic arch. Muscle thickness was measured bilaterally at rest and during gentle maximum clenching. Thickness was defined as the maximum distance between the superficial and deep layers of the temporal fascia.

**Figure 1:**
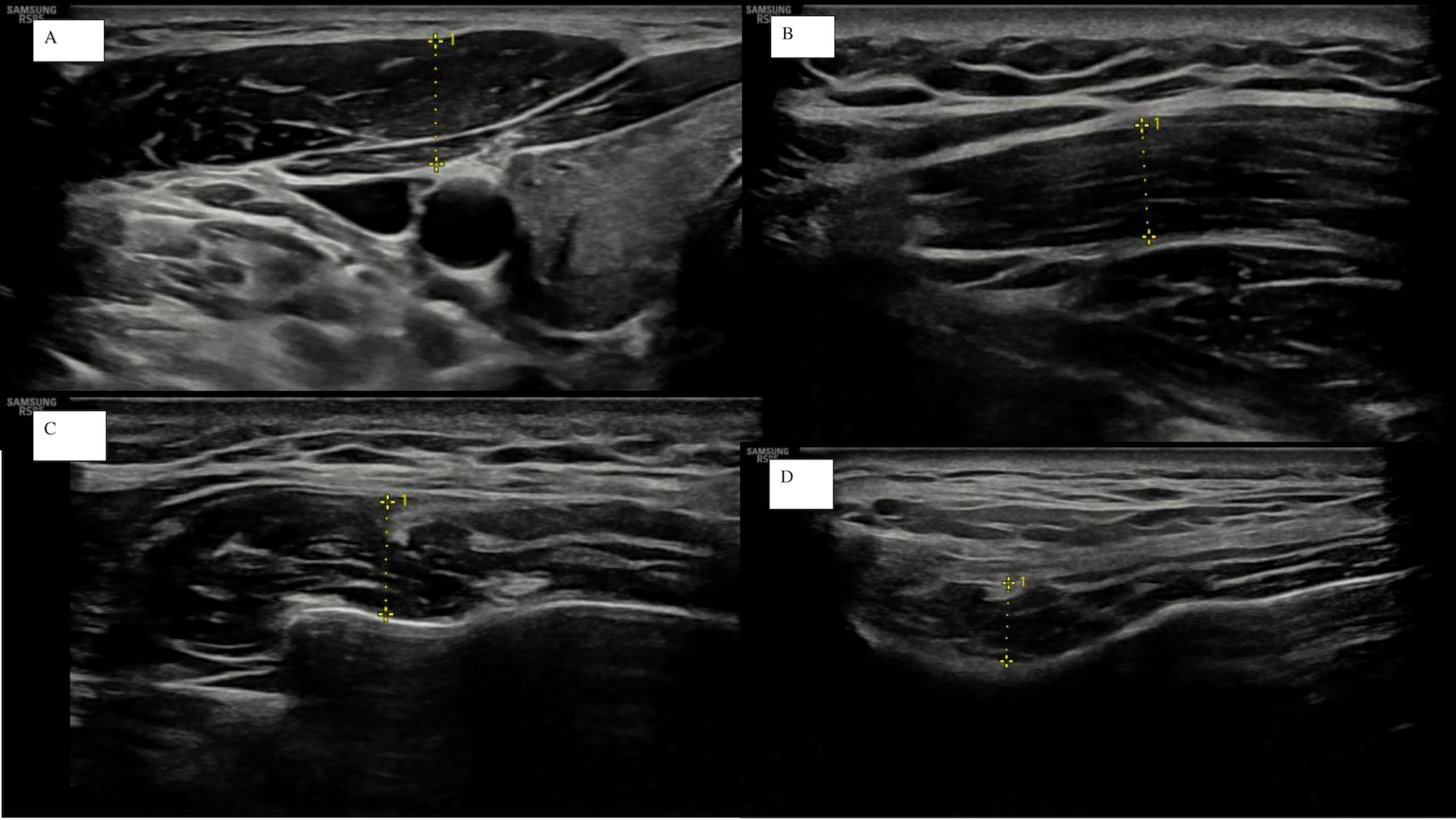
Representative ultrasonographic images of the measured muscles. (A) Sternocleidomastoid, (B) upper trapezius, (C) masseter, and (D) temporalis. Measurement lines indicate muscle thickness defined as the distance between superficial and deep fasciae.

All measurements were expressed in millimeters (mm) (Pirri et al., 2020, 2021). Representative ultrasonographic images of the evaluated muscles are shown in Fig. 1.

### Clinical assessment

Headache severity was assessed using the Visual Analog Scale (VAS), with values ranging from 0 (no pain) to 10 (worst imaginable pain) (Arikan & Çoban, 2025). Participants were specifically instructed to rate their average headache-related pain intensity over the previous 7 days, independent of the ultrasound procedure, in order to capture their baseline clinical condition.

Functional disability was evaluated using the Neck Disability Index (NDI), a validated 10-item questionnaire assessing the impact of cervical spine dysfunction on daily activities. Scores range from 0 to 50 and are interpreted as follows: 0–4 (no disability), 5–14 (mild), 15–24 (moderate), 25–34 (severe), and ≥35 (complete disability) (Elsayed & Alowa, 2025).

All clinical assessments were conducted on the same day as the ultrasound examination by the same blinded assessor to ensure consistency.

#### Statistical methods

Statistical analyses were performed using the Statistical Package for the Social Sciences (SPSS), version 25.0 (IBM Corp., Armonk, NY, USA). The normality of numerical data distribution was tested using the Kolmogorov–Smirnov and Shapiro–Wilk tests. Normally distributed continuous variables were presented as mean ± standard deviation (SD), while non-normally distributed data were expressed as median and interquartile range (IQR, 25th–75th percentiles). Qualitative variables were expressed as frequencies and percentages.

For group comparisons, the independent samples t-test was used for normally distributed continuous variables, and the Mann–Whitney U test for non-parametric data. Pearson’s chi-square (χ²) test or Fisher’s exact test was applied for categorical variables, as appropriate.

Effect sizes were calculated for between-group differences using Cohen’s d for parametric tests and r for non-parametric tests, in order to complement *p*-values with an estimate of the magnitude of observed effects. This approach was chosen to address the clinical relevance of statistically significant findings.

Spearman’s rank correlation coefficient was used to explore associations between muscle thickness, clinical outcomes (VAS and NDI), and bilateral muscle measures. Correlation analyses were performed to investigate whether changes in muscle thickness are related to symptom severity and disability, as well as to assess the internal consistency of bilateral muscle measurements.

A 95% confidence interval (CI) was applied, and a two-tailed *p*-value <0.05 was considered statistically significant.

## Results

There were no significant differences between the CGH and control groups in terms of age, gender, or body mass index (BMI) (all *p* > 0.05). The median VAS score in the CGH group was 8.0 (IQR: 7–9), and the mean NDI score was 23.7 ± 8.2, indicating a moderate degree of disability (Table 1).

**Table 1:** Demographic and clinical features of groups (*n* = 74).

	Cervicogenic headache group (*n* = 37)	Control group (*n* = 37)	*P*
	mean ± SD or median (IQR)		
Age (years)	33.6 ± 7.8	33.5 ± 7.8	1.000
Gender (*n*/%) Female Male	26 (70.3) 11 (29.7)	26 (70.3) 11 (29.7)	1.000
BMI (kg/m²)	25.0 ± 3.4	26.1 ± 4.6	0.752
Painful side (*n*/%) Right Left	21 (56.8) 16 (43.2)	–	
Duration of pain (months)	12.0 (33.0)	–	
VAS (0–10)	8.0 (1.5)	–	
NDI (0–50)	23.7 ± 8.2	–	

**Note:**

BMI, Body mass index; VAS, Visual analog scale; NDI, Neck disability index.

### Muscle thickness comparisons

Between-group analyses showed that bilateral SCM and right UT muscle thicknesses were significantly lower in the CGH group compared with controls (SCM-R: 9.4 ± 1.7 *vs*. 10.2 ± 1.3 mm, *p* = 0.016; SCM-L: 9.6 ± 1.5 *vs*. 10.2 ± 2.0 mm, *p* < 0.01; UT-R: 7.5 ± 1.6 *vs*. 8.2 ± 1.9 mm, *p* < 0.001). Effect sizes for these differences ranged from small to moderate (Cohen’s d = 0.32–0.45). No significant differences were observed in the left UT, temporalis, or masseter muscles (all *p* > 0.05) (Table 2).

**Table 2:** Comparison of muscle thickness between cervigogenic headache and control groups (*n* = 74).

Muscles	Cervicogenic headache group (*n* = 37) mean ± SD	Control group (*n* = 37) mean ± SD	*P*
Temporalis (mm)	16.2 ± 3.8	16.9 ± 2.5	0.323
Right	16.4 (2.6)	16.9 ± 2.4	0.506
Left			
Masseter (mm)	11.2 ± 2.0	11.0 ± 1.7	0.522
Right	11.2 ± 2.0	11.6 ± 1.7	0.380
Left			
Sternoclidomastoid (mm)	9.4 ± 1.7	10.2 ± 1.3	**0.016**
Right	9.6 ± 1.5	10.2 ± 2.0	**<0.01**
Left			
Trapezius (mm)	7.5 ± 1.6	8.2 ± 1.9	**<0.001**
Right	7.9 ± 1.3	8.3 ± 1.1	0.237
Left			

**Note:**

The bold values indicate statistically significant differences (*p* < 0.05).

When participants were stratified according to pain laterality (right- *vs*. left-sided CGH), no significant asymmetries were detected between the painful and non-painful sides in any muscle group (all *p* > 0.05) (Table 3).

**Table 3:** The muscle thickness according to the side of pain in the cervicogenic headache group (*n* = 37).

	Temporalis (R) (mm)	Temporalis (L) (mm)	Masseter (R) (mm)	Masseter (L) (mm)	SCM (R) (mm)	SCM (L) (mm)	Trapezius (R) (mm)	Trapezius (L) (mm)
Right-sided pain (*n* = 21)	15.7 ± 3.9	16.2 ± 3.6	10.8 ± 1.7	10.8 ± 1.8	9.4 ± 1.4	9.7 ± 1.6	7.4 ± 1.5	7.8 ± 1.7
Left-sided pain (*n* = 16)	16.7 ± 3.6	16.3 ± 3.8	11.8 ± 2.3	11.8 ± 2.2	9.3 ± 2.0	9.5 ± 1.3	7.5 ± 1.8	8.0 ± 1.8
*p*	0.437	0.743	0.123	0.138	0.793	0.719	0.889	0.801

**Note:**

R, Right; L, Left; SCM, Sternocleidomastoid muscle.

### Correlation analyses

Correlation analyses within the CGH group (Table 4) revealed:

- VAS scores were not significantly correlated with any muscle thickness (all *p* > 0.05).
- NDI scores demonstrated a moderate negative correlation with right UT thickness (rho = −0.441, *p* < 0.01), indicating that higher disability was associated with thinner UT muscle.
- A weaker but significant negative correlation was also observed between NDI and left UT thickness (rho = −0.337, *p* = 0.041).
- No significant associations were found between NDI and SCM or craniofacial muscle thicknesses.
- As expected, strong positive correlations were observed between right and left sides of each muscle (all *p* < 0.001), confirming internal consistency of bilateral morphology (Table 4).

**Table 4:** Correlation analysis of the visual analog scale and neck disability index scores with muscle thickness in the cervicogenic headache group (*n* = 37).

		Temporalis (R)	Temporalis (L)	Masseter (R)	Masseter (L)	SCM (R)	SCM (L)	Trapezius (R)	Trapezius (L)
VAS	Rho	0.255	0.213	−0.273	−0.167	−0.055	−0.002	−0.220	−0.140
	*P*	0.128	0.206	0.103	0.324	0.745	0.992	0.191	0.408
NDI	Rho	0.063	0.023	−0.269	−0.330	−0.293	−0.261	**−0.441**	−0.337
	*P*	0.712	0.893	0.108	0.056	0.078	0.119	**<0.01** **	0.041*

**Notes:**

* *p* < 0.05.

** *p* < 0.01.

Bold values indicate statistically significant correlations (*p* < 0.05).

R, Right; L, Left; VAS, Visual analog Scale; NDI, Neck disability index; SCM, Sternocleidomastoid muscle.

## Discussion

This study evaluated the structural characteristics of cervical and craniofacial muscles in individuals with CGH using USG. The main findings were significantly reduced thickness of the bilateral SCM and the right UT muscles in the CGH group compared to healthy controls, while no differences were detected in the masseter or temporalis muscles. These results are in line with prior research showing morphological alterations in cervical muscles among individuals with CGH and other neck pain disorders (Abaspour et al., 2015; Uthaikhup et al., 2017; Jull et al., 2007). The SCM and UT are superficial muscles with key roles in head posture and scapulocervical function, and their reduced thickness may reflect chronic adaptations to pain, disuse, or altered motor control rather than a direct causal mechanism (Lin et al., 2022; Falla & Farina, 2005).

Our findings are consistent with studies that have reported decreased cross-sectional area or thickness of cervical musculature in CGH populations. Reduced longus colli cross-sectional area has been demonstrated in CGH patients (Abaspour et al., 2015). In addition, age-related structural alterations in cervical muscles—such as reductions in muscle mass, decreased muscle thickness, and potential fatty infiltration—have been observed in elderly women with CGH (Uthaikhup et al., 2017). Beyond morphological findings, cervical musculoskeletal impairments including decreased endurance of deep cervical flexors, impaired proprioceptive acuity, and altered neuromuscular control strategies have been identified in patients with recurrent cervicogenic headaches (Jull et al., 2007). To our knowledge, minimal detectable change (MDC) or minimum clinically important difference (MCID) values for ultrasound-assessed cervical and craniofacial muscle thickness in individuals with CGH have not been clearly established. Therefore, the relatively small between-group differences observed in the present study (0.6–0.8 mm) should be interpreted with caution. Although ultrasonography is considered a reliable and reproducible method when standardized protocols are applied, measurement variability related to probe positioning, tissue compression, and operator dependency cannot be entirely excluded. Future studies incorporating reliability analyses and longitudinal designs are needed to better determine the clinical relevance of these morphological differences (Fernández-de-las-Peñas & Cuadrado, 2014; Biondi, 2005).

In this study, no significant differences were observed in the thickness of the masseter and temporalis muscles between individuals with CGH and healthy controls. Previous studies investigating the craniofacial musculature in headache disorders have yielded inconsistent findings. Consistent with our results, Álvarez-Méndez et al. (2017) reported no definitive relationship between masseter or temporalis thickness and pain intensity when assessed by USG. Given that CGH is primarily a cervical musculoskeletal disorder and that the trigeminal nerve innervates the masticatory muscles rather than the cervical musculature, the absence of significant differences in masseter and temporalis muscle thickness in our cohort is not unexpected. Taken together, these findings suggest that, in the absence of concomitant temporomandibular dysfunction, craniofacial muscle morphology may not be substantially altered in individuals with CGH (Manfredini et al., 2011; Svensson & Graven-Nielsen, 2001).

Interestingly, no significant correlations were found between headache severity (VAS scores) and muscle thickness, suggesting that subjective pain intensity in CGH may not be directly explained by peripheral muscle morphology. Instead, pain perception in CGH is likely influenced by complex mechanisms such as central sensitization, altered sensorimotor control, and postural strain (Abaspour et al., 2015; Uthaikhup et al., 2017). Consistent with this interpretation, Onan et al. (2024) reported an association between reduced multifidus muscle thickness and higher pain intensity in chronic neck pain patients, whereas no clear correlation was found for superficial muscles such as the SCM or trapezius. Similarly, Lin et al. (2022) demonstrated that trapezius muscle thickness was not significantly related to pain scores, but changes in muscle elasticity were more closely linked with pain intensity. These findings suggest that deep cervical musculature and neuromuscular properties other than thickness may have a more substantial role in the the clinical presentation of CGH.

In this study, a significant negative correlation was observed between UT muscle thickness and NDI scores, indicating that reduced muscle thickness was associated with greater disability. This finding is consistent with previous reports linking morphological changes in cervical musculature with impaired function. For example, Güneş et al. (2025) investigated the relationship between forward head posture, cervical muscle thickness, and neck pain-related disability, reporting that muscle morphology and disability may be related. Similarly, Van der Linden (2019) described an association between decreased trapezius and SCM thickness and greater disability in patients with chronic neck pain. Karabaş et al. (2022) also reported that higher NDI scores correlated with reduced trapezius thickness and functional limitations. Collectively, these findings suggest an association between cervical muscle morphology and functional impairment in individuals with CGH.

Taken together, our results highlight the potential importance of cervical muscle integrity in the clinical presentation of CGH. Interventions targeting the strengthening and functional activation of superficial cervical muscles, particularly the SCM and trapezius, may help improve postural stability and reduce disability, although causality cannot be established based on the present cross-sectional data. USG appears to be a feasible, non-invasive method for monitoring muscle morphology in this population, and its role as an adjunct for evaluating treatment outcomes warrants further longitudinal research rather than immediate diagnostic application (Uthaikhup et al., 2017).

This study has several limitations that should be acknowledged when interpreting the findings. First, the relatively small sample size and single-center design may restrict the generalizability of the results. Although groups were age-and sex-matched, larger multicenter studies with more diverse populations are needed to enhance external validity.

Second, this study focused exclusively on muscle thickness as a morphological parameter. Functional and biomechanical properties such as stiffness, elasticity, and neuromuscular control were not assessed. Third, the cross-sectional design prevents causal inferences. It cannot be determined whether the observed muscle thinning represents a predisposing factor or a secondary consequence of CGH. Longitudinal studies are required to clarify temporal relationships and evaluate adaptations over time or following rehabilitation interventions. Occupational characteristics, hand dominance, and habitual physical activity levels were not systematically evaluated in the present study. As these factors may influence cervical and craniofacial muscle morphology, their potential confounding effects cannot be fully excluded.

Finally, only VAS and NDI were used as clinical outcomes. While these instruments are widely validated, they provide subjective measures of pain and disability. The lack of complementary assessments such as surface electromyography (sEMG), postural analysis, and proprioceptive testing limited the scope of neuromuscular evaluation. Future research integrating these modalities would provide a more comprehensive understanding of the complex pathophysiology of CGH.

In conclusion, individuals with cervicogenic headache exhibited reduced thickness of the SCM and right UT muscles compared with healthy controls, whereas no significant differences were observed in the masseter or temporalis muscles. These findings indicate that superficial cervical muscle morphology may differ in individuals with CGH; however, the relatively small between-group differences warrant cautious interpretation regarding their clinical relevance. USG proved to be a feasible and non-invasive method for assessing cervical and craniofacial muscle thickness in this population. While it may serve as a useful adjunct in clinical evaluation, longitudinal and interventional studies are needed before any diagnostic or prognostic implications can be inferred. From a rehabilitation perspective, assessment and targeted management of both superficial and deep cervical muscles—particularly when deficiencies are identified—may be considered within comprehensive treatment programs. Future research integrating structural and functional assessments will further clarify how muscle morphology relates to clinical presentation in CGH.

## Supplemental Information

10.7717/peerj.21285/supp-1 Supplemental Information 1 The complete dataset used in the study, including all variables and measurements analyzed for statistical evaluation.

10.7717/peerj.21285/supp-2 Supplemental Information 2 STROBE Checklist.

10.7717/peerj.21285/supp-3 Supplemental Information 3 Anonymised raw data for the control group in English.

10.7717/peerj.21285/supp-4 Supplemental Information 4 Codebook.

10.7717/peerj.21285/supp-5 Supplemental Information 5 Ultrasonographic assessment of cervical and craniofacial muscle thickness in cerviogenic headache.

## Additional Information and Declarations

### Data Availability

The following information was supplied regarding data availability:

The data is available in the Supplemental Files.

## References

[ref-2] Abaspour O, Akbari M, Rezasoltani A (2020). Ultrasonography method of deep cervical muscles and thickness measurement reliability in cervicogenic headache and healthy subjects: a pilot study. Journal of Modern Rehabilitation.

[ref-3] Abaspour O, Javanshir K, Amiri M, Karimlou M (2015). Relationship between cross sectional area of Longus Colli muscle and pain laterality in patients with cervicogenic headache. Journal of Back and Musculoskeletal Rehabilitation.

[ref-4] Álvarez-Méndez AM, Exposto FG, Castrillon EE, Svensson P (2017). Systematic mapping of pressure pain thresholds of the masseter and temporalis muscles and assessment of their diversity through the novel application of entropy. Journal of Oral & Facial Pain and Headache.

[ref-5] Arikan H, Çoban T (2025). Low back activity confidence scale: cross-cultural adaptation, reliability, and validity of the Turkish version in individuals with non-specific low back pain. Journal of Back and Musculoskeletal Rehabilitation.

[ref-6] Biondi DM (2005). Cervicogenic headache: a review of diagnostic and treatment strategies. Journal of Osteopathic Medicine.

[ref-7] Choi W (2021). Effect of 4 weeks of cervical deep muscle flexion exercise on headache and sleep disorder in patients with tension headache and forward head posture. International Journal of Environmental Research and Public Health.

[ref-8] Edvinsson JCA, Viganò A, Alekseeva A, Alieva E, Arruda R, De Luca C, D’Ettore N, Frattale I, Kurnukhina M, Macerola N, Malenkova E, Maiorova M, Novikova A, Řehulka P, Rapaccini V, Roshchina O, Vanderschueren G, Zvaune L, Andreou AP, Haanes KA (2020). The fifth cranial nerve in headaches. The Journal of Headache and Pain.

[ref-9] Elsayed WH, Alowa ZA (2025). Impact of forward head posture correction on craniovertebral angle, neck disability, and spinal electromyography: a randomized controlled trial. Journal of Back and Musculoskeletal Rehabilitation.

[ref-10] Falla D, Farina D (2005). Muscle fiber conduction velocity of the upper trapezius muscle during dynamic contraction of the upper limb in patients with chronic neck pain. Pain.

[ref-11] Fernández-de-las-Peñas C, Cuadrado ML (2014). Therapeutic options for cervicogenic headache. Expert Review of Neurotherapeutics.

[ref-31] Güneş S, Şahinkaya Ş, Yamak S, Kaplan S, Sarikaya AR, Demirhan OE, Kar I, Kutlay S (2025). Relationship of forward head posture with cervical muscle thickness and neck pain-related disability among young adults: a cross-sectional study. International Journal of Osteopathic Medicine.

[ref-1] International Headache Society (IHS) (2018). Headache classification committee of the International Headache Society (IHS) the international classification of headache disorders, 3rd edition. Cephalalgia.

[ref-12] Jull G, Amiri M, Bullock-Saxton J, Darnell R, Lander C (2007). Cervical musculoskeletal impairment in frequent intermittent headache. Part 1: subjects with single headaches. Cephalalgia.

[ref-13] Karabaş Ç, Aras B, Erol K, Kuzu Ö (2022). Sonographic comparison of neck extensor muscle thickness of ankylosing spondylitis and non-radiographic axial spondyloarthritis patients with healthy volunteers. Aktuelle Rheumatol.

[ref-14] Lin LZ, Yu YN, Fan JC, Guo PW, Xia CF, Geng X, Zhang SY, Yuan XZ (2022). Increased stiffness of the superficial cervical extensor muscles in patients with cervicogenic headache: a study using shear wave elastography. Frontiers in Neurology.

[ref-15] Manfredini D, Guarda-Nardini L, Winocur E, Piccotti F, Ahlberg J, Lobbezoo F (2011). Research diagnostic criteria for temporomandibular disorders: a systematic review of axis I epidemiologic findings. Oral Surgery, Oral Medicine, Oral Pathology, Oral Radiology, and Endodontology.

[ref-16] Mohammadi Z, Shafizadegan Z, Tarrahi MJ, Taheri N (2021). The effectiveness of sternocleidomastoid muscle dry needling in patients with cervicogenic headache. Advanced Biomedical Research.

[ref-17] Onan D, Demirci E, Turhan E, Ülger Ö (2024). Spinal stabilization exercises for transversus abdominis and lumbar multifidus thickness via telerehabilitation and face-to-face approaches in patients with nonspecific chronic neck pain: a randomized controlled trial. Turkish Journal of Medical Sciences.

[ref-18] Peterson G, Leary SO, Nilsson D, Moodie K, Tucker K, Trygg J, Peolsson A (2019). Ultrasound imaging of dorsal neck muscles with speckle tracking analyses-the relationship between muscle deformation and force. Scientific Reports.

[ref-19] Pirri C, Fede C, Fan C, Guidolin D, Macchi V, De Caro R, Stecco C (2021). Ultrasound imaging of head/neck muscles and their fasciae: an observational study. Frontiers in Rehabilitation Sciences.

[ref-20] Pirri C, Stecco C, Fede C, Macchi V, Özçakar L (2020). Ultrasound imaging of the fascial layers: you see (Only) What you know. Journal of Ultrasound in Medicine.

[ref-21] Sedlackova Z, Vita M, Herman J, Furst T, Dornak T, Herman M (2022). Elasticity of neck muscles in cervicogenic headache. Biomedical Papers of the Medical Faculty of the University Palacký, Olomouc, Czech Republic.

[ref-22] Svensson P, Graven-Nielsen T (2001). Craniofacial muscle pain: review of mechanisms and clinical manifestations. Journal of Orofacial Pain.

[ref-23] Uthaikhup S, Assapun J, Kothan S, Watcharasaksilp K, Elliott JM (2017). Structural changes of the cervical muscles in elder women with cervicogenic headache. Musculoskeletal Science and Practice.

[ref-24] Van der Linden S (2019). A pilot study using ultrasound imaging to compare fascial thickness between chronic neck pain and control groups. https://hdl.handle.net/10652/4625.

