# Supplementary material for: Ultrasonographic assessment of cervical and craniofacial muscle thickness in individuals with and without cervicogenic headache
Source: PeerJ. 2026 May 25;14:e21285. doi: 10.7717/peerj.21285 (PMC13218338; doi:10.7717/peerj.21285)
Supplement: Supplemental Information 4 [file peerj-14-21285-s004.docx]

Codebook

Grup 0: servikojenik baş ağrısı olan grup

Grup 1: servikojenik baş ağrısı olmayan grup

Cinsiyet

0: kadın

1: erkek

Codebook

Group 0: Group with cervicogenic headache

Group 1: Group without cervicogenic headache

Gender

0: female

1: male
