# Supplementary figures and images for: Ultrasonographic assessment of cervical and craniofacial muscle thickness in individuals with and without cervicogenic headache
Source: PeerJ. 2026 May 25;14:e21285. doi: 10.7717/peerj.21285 (PMC13218338; doi:10.7717/peerj.21285)

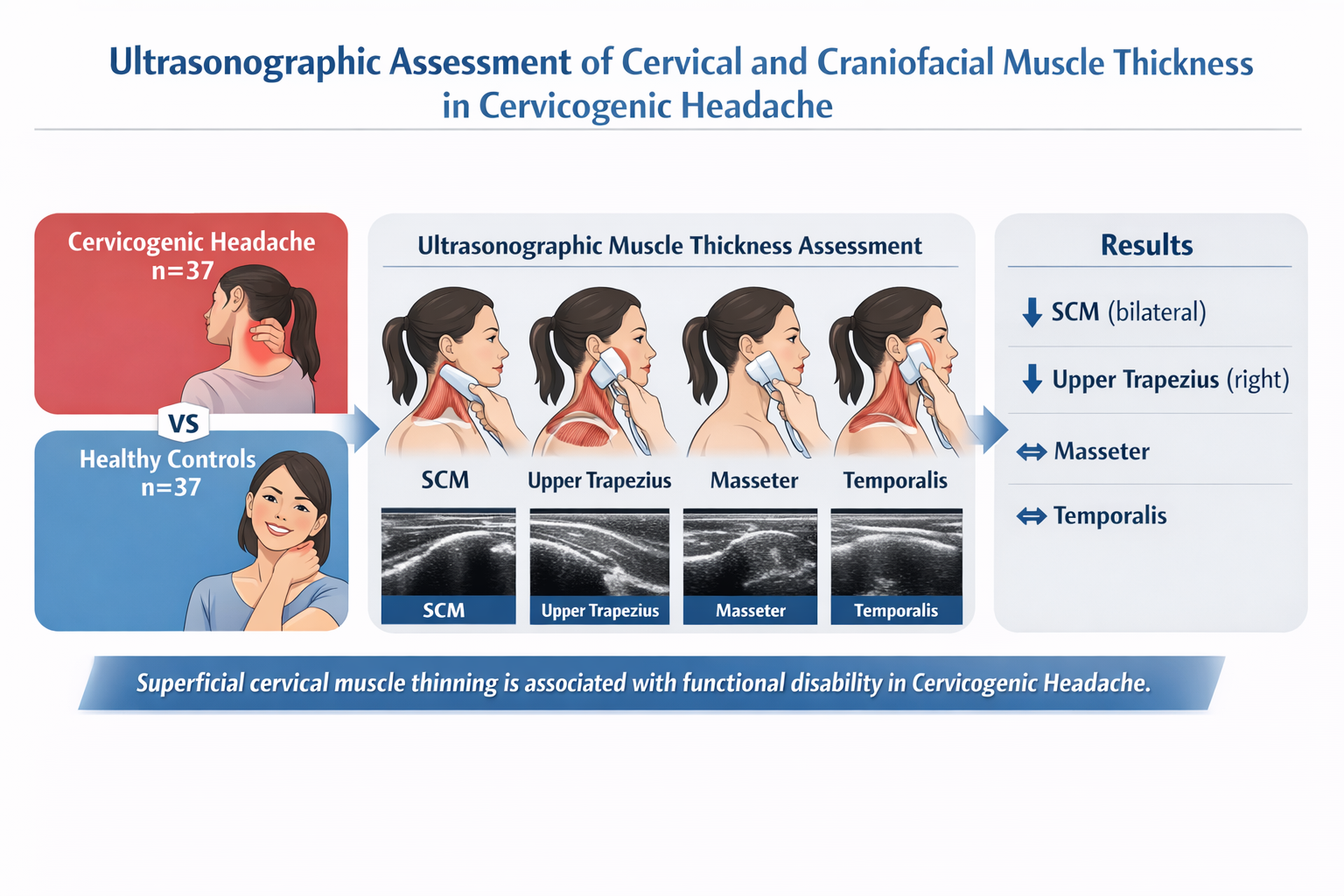

Supplement: Supplemental Information 5 [file peerj-14-21285-s005.png]
